# Analysis of the Effect of Skew Rolling Parameters on the Surface Roughness of C60 Steel Products Using ML Methods

**DOI:** 10.3390/ma16227136

**Published:** 2023-11-12

**Authors:** Konrad Lis

**Affiliations:** Department of Metal Forming, Mechanical Engineering Faculty, Lublin University of Technology, 20-618 Lublin, Poland; k.lis@pollub.pl

**Keywords:** skew rolling, C60 steel, roughness, CNC rolling mill, stepped axles and shafts, metal forming, machine learning models

## Abstract

This paper presents results from experimental and numerical studies of the skew rolling process used to shape axisymmetric products made of C60-grade steel. An experimental study was carried out to investigate the effect of process parameters described by the forming angle *α*, the skew angle *θ*, the reduction ratio *δ*, and the jaw chuck velocity *V_u_* on the surface roughness *Ra* of the forgings. Stepped forgings made of C60-grade steel were rolled. Based on numerical calculations, a machine learning regression model was developed that uses process parameters to predict the surface roughness of produced parts. The random forest model was found to be the most effective based on the determined metrics (MAE, RMSE, R^2^). A more detailed analysis of this model was performed using the SHAP library. The application of ML methods will enable optimization of skew rolling through appropriate selection of process parameters affecting improvement in product quality.

## 1. Introduction

**Figure 1:**
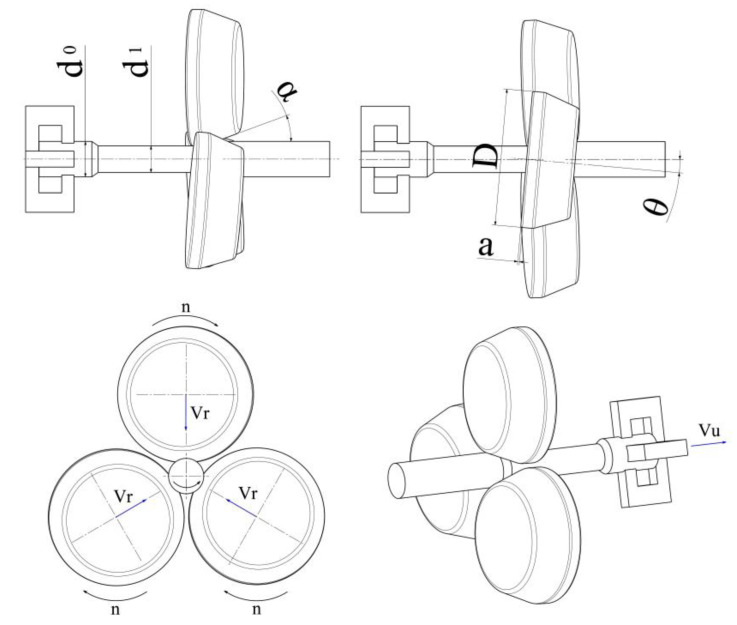
Schematic design of a skew rolling process conducted with three tapered rollers.

The skew rolling method is used to produce elongated axisymmetric parts. They can be formed either from steel or from nonferrous metal alloys [1]. A schematic design of this process is shown in Figure 1. The process is conducted with the use of three tapered rollers. They are located on the circumference of the billet, every 120°. Each roller is rotated in the same direction with the same rotational speed *n*. The rollers (each having the same diameter *D*) are described by a forming angle *α* and a calibrating section with a length *a*. They are all set askew at an angle *θ* to the rolling axis. They can diverge and converge, as a result of which the initial billet diameter *d*_0_ is reduced to the desired diameter *d*_1_. In addition to that, their velocity *V_r_* is synchronized with the chuck’s velocity *V_u_*. One end of the billet is mounted in the chuck jaws. Synchronized movements of the tapered rollers and the chuck allow for rolling parts with variable cross-sections.

Early studies were numerical analyses based on FEM. Among other things, the rolling of a shaft with a length of about 605 mm and a diameter of 56 mm (for the largest dimeter section) [2] and of a rail car axle with a length of about 2150 mm and a diameter of 202 mm (for the largest diameter section) [3] were simulated. The possibility of rolling a stepped crankshaft with a length of 491 mm and a diameter of 75 mm (for the largest diameter section) was also investigated [4]. All these parts were made of the C45-grade steel. A novel concept was developed by rolling hollow axles on a mandrel to maintain the inside diameter of the rolled part [5]. Rolling processes for solid [6,7] and hollow parts [8] made of nonferrous metal alloys were also simulated. The numerical results showed that the process was characterized by moderate force parameters in relation to rolled part dimensions. In addition, the forming time is much shorter compared to alternative manufacturing methods, which also means reduced energy consumption of this process. A major advantage of this process is that it can be used to form products with variable cross-sections using a single set of working tools.

Later studies also included experiments on producing parts by skew rolling conducted with three tapered rollers. One of these studies investigated rolling of two types of rail car axles, i.e., according to American and European standards at a scale of 1:5 [9]. The billet was a 42CrMo4 steel bar. The experimental results showed high dimensional accuracy of the rolled parts. Surface defects in the form of small helical grooves over the circumference of the rolled parts were observed. The experiments were also conducted on rolling hollow axles (made of LZ50 and 30CrMoA grades of steel), which led to the determination of optimal process parameters, ensuring the required microstructure quality [10,11]. Hollow parts were also rolled (made of 42CrMo4-grade steel) with the use of a mandrel in order to maintain their inside diameter constant [12].

Studies [13,14] presented a modification of the three-roller skew rolling method, called tandem flexible skew rolling. In this method, two-roller skew rolling is used to hollow the billet with the Mannesmann method first, and then the billet is placed on a mandrel. After that, rolling is conducted with three tapered rollers that shape the workpiece as desired. The proposed solution reduces, among other things, the area needed for the rolling mill setup, rolling forces, as well as tool manufacturing costs.

Studies also investigated the effect of skew rolling process parameters on the dimensional accuracy of cross-sections and on the surface quality of rolled parts. According to these studies, the surface roughness of tapered parts of products could be improved, among other things, by increasing the rotational speed of the working tools [15] or by modifying tool geometry [16]. These would include introducing a tapered exit zone of the rollers or increasing the rounding radius of the rollers in the exit zone. In addition, the effects of parameter combinations on the surface quality and product roundness deviations [17] were determined via ANOVA analysis. This made it possible to estimate the recommended rotational speeds and radial feeds of tapered rollers to minimize failure modes in the process.

The surface quality of rolled parts depends on the initial conditions of the rolling process. Surface quality may also have impact on further processing of preforms. This means that the initial parameters of rolling must be appropriately selected in order to produce parts with a satisfactory surface quality. This is described, among other things, by the roughness profile. The most widely used parameters describing material roughness profile are *Ra* and *Rz.* Achieving the desired roughness of a material is a complex task, one that depends on many factors. This aspect is all the more important when this rolling process is employed in forging plants, where it is used for fabricating semifinished parts for further processing. One of the effective ways to predict the final roughness value is to simulate the process using artificial intelligence-based models. They have the ability to learn and adapt to a set of input data. 

In subtractive manufacturing, turning and milling machine learning methods are predominantly used to select process parameters to achieve a satisfactory material surface. The *Ra* value of parts produced by turning was predicted using artificial neural network techniques, as described in a study [18]. Multivariate regression models were also used, yielding a high value of the coefficient of determination R^2^ [19].

Similar studies were also conducted for the milling process using regression models and artificial neural networks. The developed models were validated, showing their high fit to real values [18,20]. The *Ra* roughness was also predicted using classification methods [21]. A continuous variable (which is a result of regression) was replaced by discrete variables, enabling *Ra* parameter categorization into several value ranges. Support vector machine (SVM), k-nearest neighbours (kNN), decision tree, and random forest models were used.

In metal forming, the quality of obtained products is also analysed. Machine learning methods make it possible to automate this process. The methods have been used to predict the surface quality of sheet metal parts produced using incremental forming (specifically, single-point incremental forming, SPIF). One way involved using a classification method with the SVM model [22]. Three roughness classes were distinguished for Ti6A14V surfaces. To increase the effectiveness of the model, the training data additionally included waviness parameter values. The other solution was to use regression with, among others, artificial neural network (ANN), and support vector regression models. They were used to predict the roughness parameters *Ra* and *Rz* for parts made of the extra deep drawing (EDD)-grade steel that is widely used in the automotive industry [23]. Based on the calculated coefficient of determination R^2^, it was found that SVR showed higher efficiency.

Machine learning models were also applied in the extrusion of U-channel and square cup parts [24]. Failure modes, including the spring-back effect (U-Channel) and product wall thickness reduction (U-channel and square cup), were classified for three grades of steel: DC06, HSLA340, and DP600. Seven models were used: multilayer perceptron (MLP), decision tree, random forests, support vector machine, k-nearest neighbours, and logistic regression. Classification was performed using both single models and ensembles created from several models. For the former, the accuracy, as expressed by the F-score, was 85%. The ensemble models achieved slightly higher F-score values (about 90%) compared to the single models, yet they showed a better bias–variance trade-off.

Another example of machine learning use is the analysis of defects on the hot-rolled sheet metal surface induced due to scale formation [25,26]. Different types of material defects were classified using several models, including artificial neural networks, SVM (due to their popularity in defect analysis), and decision trees. Each of these models yielded a high value of accuracy.

Multiclass classification models, including multiclass SVM, were applied for the evaluation of hot-rolled bar surface quality [27]. Input data in the form of a set of images of five different materials additionally contained five predominant surface defects obtained with an imaging system. Initial model runs correctly detected two of the five defects. A much lower degree of recognition was obtained with the remaining three. Subsequent modifications, including those of the input data, led to increasing the SVM model’s effectiveness. This resulted in the detection of all five material defects with a satisfactory degree of accuracy.

This study presents experimental and numerical results. It was proposed to use machine learning methods to predict the quality of cylindrical parts produced using skew rolling. The literature review shows that there is a lack of comprehensive studies that employ machine learning methods to investigate metal forming processes. The objective of this study was to select a regression model that effectively predicts the *Ra* roughness value of parts made of C60-grade steel. The reason for taking up this research problem is to select parameters of this forming process that ensure the lowest possible roughness on the product surface. The obtained material surface quality can affect further forming operations, including forging. This, in turn, can be a cause of internal defect formation in forged parts, as a result of which they are classified as waste. The results make it possible to enhance the efficiency of the rolling process by reducing the time required for determining the optimal parameters of this process. 

The dataset used for training the machine learning models was taken from the experimental studies on skew rolling conducted at the Lublin University of Technology. This paper describes this forming method, the scope of the study, and discusses the obtained results. It goes on to present the proposed models and the metrics used for evaluating their accuracy. The final part of this paper offers a summary and conclusions.

## 2. Materials and Methods

Experiments on skew rolling process were conducted in compliance with the setup provided in Table 1. Three sets of tapered rollers were used with variable forming angles *α* of 15°, 20°, and 25° (each having the same length of the calibrating section *a* equal to 13 mm). Each toolset was described using an angle *θ* with respect to the rolling axis. Three tool angles *θ* were used, i.e., 2.5°, 5°, and 7.5°. In addition, for each angle setting, the test specimen was rolled using three different values of *V_u_*, i.e., 10 mm/s, 20 mm/s, and 40 mm/s. Variable cross-sectional reductions of the specimens were applied, expressed by the reduction ratio *δ* with values of 1.13, 1.3, 1.53. This quantity is described by the following equation:

(1) $$\delta =\frac{d_{0}}{d_{1}}$$

where *d*_0_ is the initial billet diameter, *d*_1_ is the reduced diameter. The tapered rollers were rotated at a constant speed *n*, equal to 60 rpm. Samples of C60-grade steel with a diameter of 52 mm and a length of 330 mm were rolled. The chemical composition and mechanical properties of the material are provided in Table 2 and Table 3.

### 2.1. Test Stand and Experimental Tests

**Figure 2:**
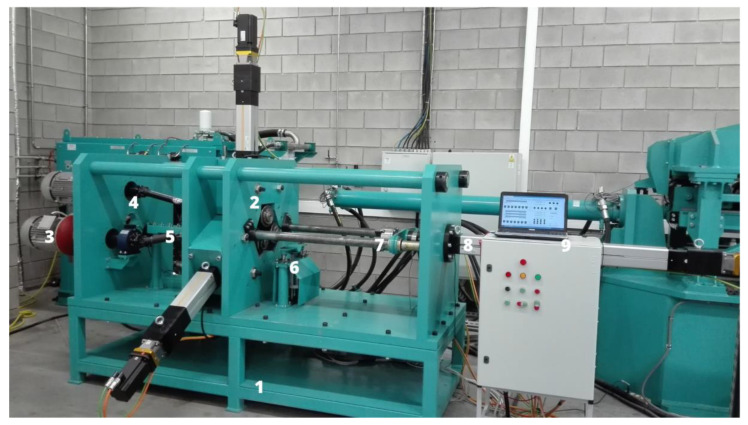
Laboratory skew rolling mill (described in the text).

The experimental test stand used in this study is shown in Figure 2. The design requirements of the rolling mill were based on the results of numerical simulations for stepped axes and shafts, as described in the previous section. These were used in combination with FEM analysis results [28], which provided data about force parameters during rolling for variable setting parameters.

The laboratory skew rolling mill consisted of segments [29]. It consisted of a support frame (1), mill stand (2), drive unit (3), power transmission system (4), billet support (5) and finished product support units (6), jaw chuck unit (7), and axial cylinder unit (8). The test stand was equipped with a measuring system and numerical control (9). The latter was based on the PAC controller for controlling the drivers of individual electro-screw actuators, i.e., 3 radial (working tools) and 1 axial (jaw chuck). The HMI panel was provided via a portable computer. It allowed for ongoing analysis of the rolling mill parameters during the forming process and was used to upload a program defining actuator motion. It was necessary to upload a file with the specified envelope of the final product. Dedicated software was used to convert it to a command line in the form of G-code, which was already an execution file. As a result, the operation of the tapered rollers and the jaw chuck could be synchronized.

The proposed numerical control makes it possible to roll axisymmetric products of different shapes and lengths using the same set of tools. Consequently, the process can already become cost-effective in the case of unit production. This method primarily reduces the costs of manufacturing complex geometry tools. 

**Figure 3:**
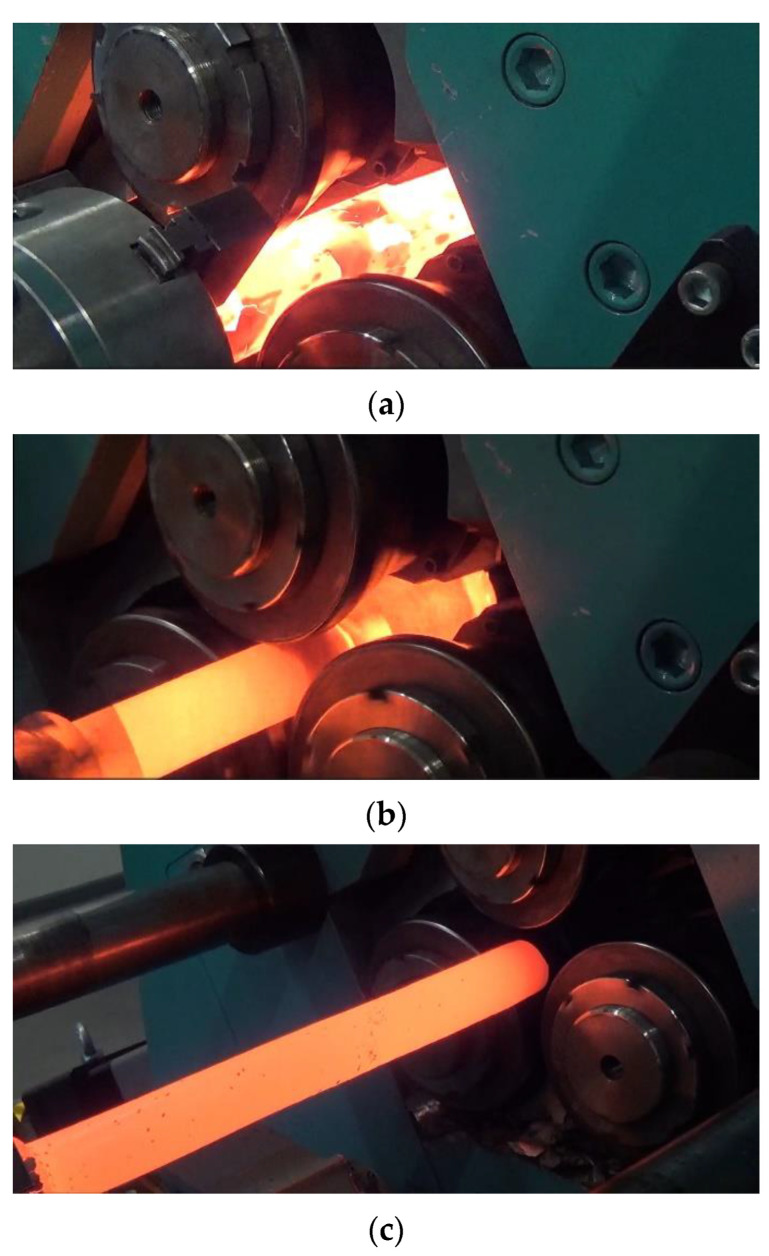
Stages of the analysed rolling process: (**a**) beginning of the process, (**b**) middle of the process, (**c**) end of the process.

Experiments were carried out on a laboratory skew rolling mill. Prior to rolling, the billet (a rod made of C60 steel) was heated to 1200 °C in an electric chamber furnace. After that, it was mounted in a jaw chuck and rolled (according to the setup provided in Table 1). The stages of the process are shown in Figure 3. 

After that, the roughness of the outer surface of the rolled parts was measured. For the evaluation of material surface quality, the parameter *Ra* was used. This parameter is defined as the arithmetic mean of the ordinates of the *y(x)* profile inside the elementary length *l* calculated according to Equation (2). It can also be presented in the form of an approximate formula (3). The measurement was made with the contact method using Hommel-Etamic’s 3D T8000 RC120-400 with a diamond measuring tip. The measuring length was set to 48 mm and the measuring tip speed was 1 mm/s.

(2) $$R_{a}=\frac{1}{l}\int_{0}^{l}\left|y(x)\right|\;dx$$

(3) $$R_{a}=\frac{1}{n}\sum_{i=1}^{n}\left|y_{i}\right|$$

### 2.2. Numerical Modelling

Machine learning models were used to predict the *Ra* roughness parameter of the steel samples. Based on previous studies, the following models were selected: linear and polynomial regressors, random forest regressor, support vector regression, and XGBoost. The first four were taken from the scikit-learn library [30], while the last one was obtained from XGBoost [31]. Regression models were used to predict the continuous value.

A dataset was created based on the setting parameters of the rolling process (Table 1) and the experimental material roughness results. The input variables for the model were the forming angle *α* (15°, 20°, 25°), the tool angle *θ* (2.5°, 5°, 7.5°), the axial velocity of the chuck *V_u_* (10 mm/s, 20 mm/s, 40 mm/s), and the reduction ratio *δ* (1.13, 1.3, 1.53). The predicted value was the *Ra* parameter (continuous value) expressed in μm. The dataset was divided into a training set (containing 75% of the entire set) and a test set (25% of the entire set). Calculations were carried out in the Google Colaboratory environment.

For the determination of model effectiveness, the following model evaluation metrics were employed: coefficient of determination (R^2^), mean absolute error (MAE), root mean square error (RMSE). They are described by the following equations:

(4) $$R^{2}=1-\frac{\sum_{i=1}^{n}{\left(y_{i}-{\hat{y}}_{i}\right)}^{2}}{\sum_{i=1}^{n}{\left(y_{i}-\bar{y}\right)}^{2}}$$

(5) $$MAE=\frac{1}{n}\sum_{i=1}^{n}\left|y_{i}-{\hat{y}}_{i}\right|$$

(6) $$RMSE=\sqrt{\frac{\sum_{i=1}^{n}{\left(y_{i}-{\hat{y}}_{i}\right)}^{2}}{n}}$$

where:

$y_{i}$—i-th observation of the *y* variable;
${\hat{y}}_{i}$—theoretical value of the variable based on a model;
$\bar{y}$—arithmetic mean of the experimental values of the variable;
*n*—number of the samples of the variable.

The input variables were standardised to ensure data uniformity using StandardScaler from the scikit-learn library. The standardisation was conducted in compliance with the formula below:

(7) $$z=\frac{x-\mu }{\sigma }$$

*x*—experimental value of the input variable;
*μ*—arithmetic mean of the input data for a given parameter;
*σ*—standard deviation of the input data for a given parameter.

The GridSearchCV method (from the scikit-learn library) was used to select the hyperparameters of selected regression models. The method allows for increasing the degree of fit of predicted values to measured values. A cross-validation method was employed to verify that there was no overtraining of the models. This involved dividing the training set into smaller subsets. After that, one of these subsets was selected and treated as a test set. By repeating the process *k*-times (depending on the number of subsets), it was possible to calculate the selected model evaluation metric each time.

## 3. Results and Discussion

### 3.1. Surface Roughness

**Figure 4:**
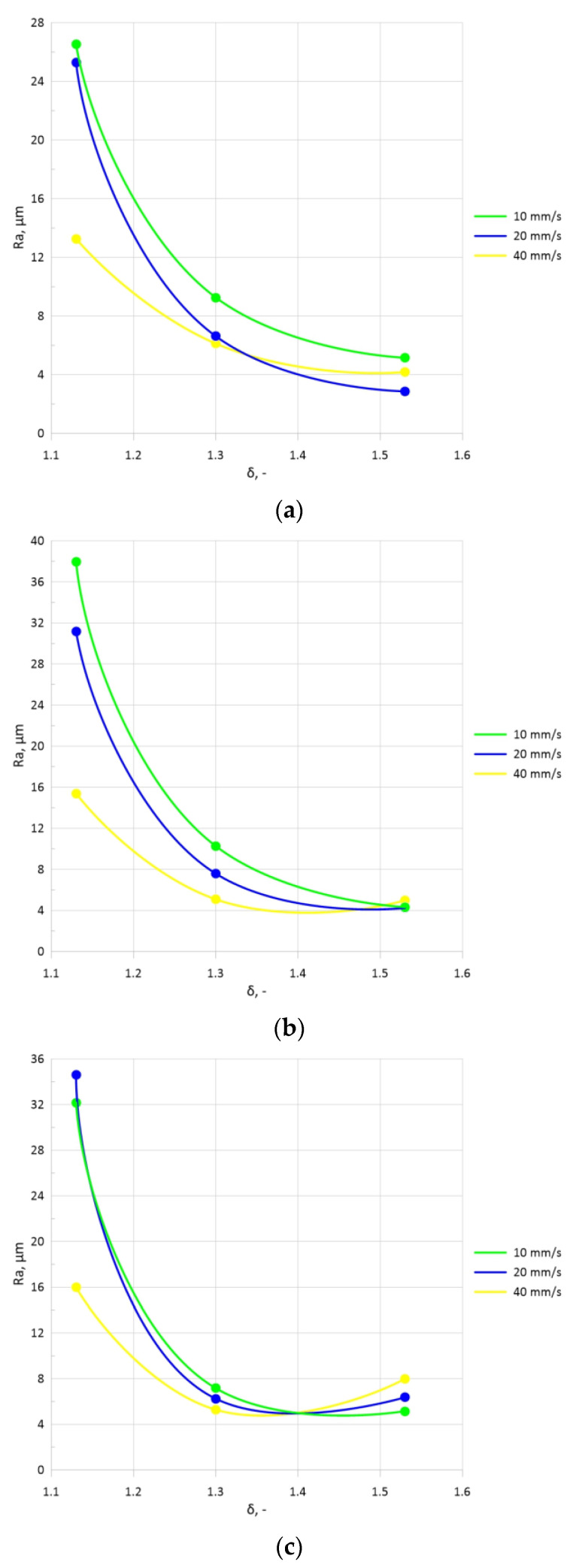
Roughness parameter *Ra* of parts rolled with the use of tapered rolls described by *θ* = 5°: (**a**) *α* = 15°, (**b**) *α* = 20°, (**c**) *α* = 25°.

Based on the material surface layer examination, it was determined that increasing the forming angle *α*, predominantly for *θ* = 5° and 7.5°, resulted in an increase in the *Ra* roughness of samples made of C60-grade steel. When the *α* angle was increased from 15° to 20°, the *Ra* value increased by more than 25% on average and by 18% when the *α* angle was increased from 15° to 25°. In addition to that, an increase in the tool angle *θ* resulted in increased surface roughness. When the *θ* angle was increased from 2.5° to 5°, the *Ra* value increased by 56% on average. When the angle was increased from 5° to 7.5°, the parameter value increased by 23%. Selected results of roughness versus *V_u_* are shown in Figure 4. For a more accurate visualization of the changes, dot diagrams connected by lines were plotted (intermediate values are not shown). They only refer to the tapered rollers that were set askew to the rolling axis at an angle *θ* equal to 5°.

**Figure 5:**
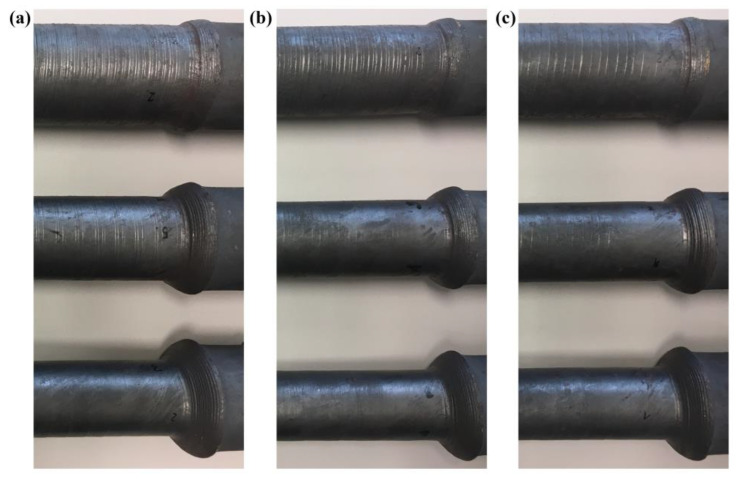
Outer surface of parts formed with the use of tapered rollers described by *α* = 20° (*θ* = 5°) and the reduction ratio increased from *δ* = 1.13 to *δ* = 1.53 (starting from top): (**a**) *Vu* = 10 mm/s, (**b**) *Vu* = 20 mm/s, (**c**) *Vu* = 40 mm/s.

It was observed that *V_u_* affected the roughness parameter. In the process conducted with the lowest speed, i.e., 10 mm/s, the *Ra* values were the highest. An increase in *V_u_* led to reduced roughness, which resulted in a higher quality of the outer surface of the rolled parts. In addition, an increase in the reduction ratio resulted in reduced roughness values. The highest values were obtained with *δ* = 1.13. The results demonstrated that higher velocities *V_u_* should be used for smaller reduction ratios, i.e., when *δ* ≤ 1.13. For higher reduction ratios (*δ ≥* 1.3), the chuck velocity *V_u_* should be at least twice lower than the axial speed of the roll resulting from the askew positioning of the working tools. Figure 5 also shows the obtained outer surfaces of the rolled parts for the abovementioned setting parameters.

### 3.2. Numerical Modelling

**Figure 6:**
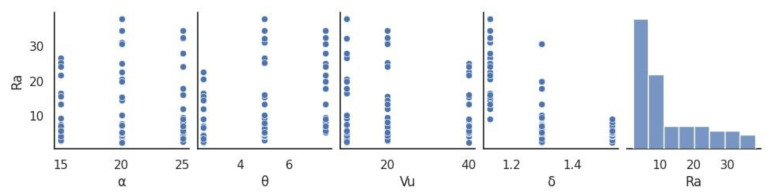
Dot diagrams illustrating the relationship between roughness value and rolling parameters.

The prepared dataset was used to generate dot diagrams (Figure 6) illustrating the relationship between individual parameters of the skew rolling process and the roughness *Ra* value.

The experimental results shown in the diagrams provide more insight into the effects of the input data on the *Ra* parameter. An increase in the forming angle *α* causes an increase in the surface roughness of products. A similar relationship can be observed for the tool angle *θ*. The use of the *θ* angle of 5° and 7.5° results in higher *Ra* values. An increase in the chuck velocity *V_u_* led to reduced surface roughness of the samples. The use of a higher diameter reduction (higher *δ*) also improved their surface quality of the products. The diagrams also contain histograms (of one-sided type) with a frequency distribution of the roughness values.

The calculations made for the test set made it possible to evaluate the effectiveness of the selected machine learning models. The following metrics were used to that end: mean absolute error (MAE), root mean square error (RMSE), and the coefficient of determination (R^2^). Obtained results are listed in Table 4.

**Figure 7:**
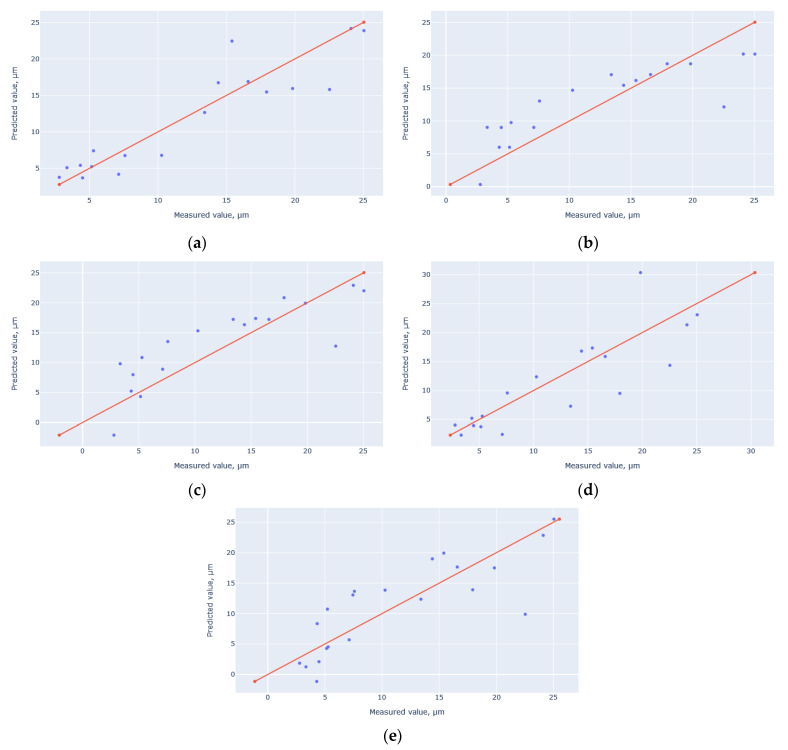
Model fitting: (**a**) random forest, (**b**) support vector machine, (**c**) XGBoost, (**d**) linear regression, (**e**) polynomial regression.

The random forest model showed the highest fit of the predicted results to the measured values of *Ra*, yielding the R^2^ coefficient value of 0.84. This can also be observed in the diagram in Figure 7a, where these values are located the closest to the straight line that cuts the diagram into two parts. The model is also characterized by the lowest MAE value of 2.29 μm and the RMSE value of 2.98 μm. The other models showed lower fit, ranging from R^2^ = 0.69 for the SVM model to R^2^ = 0.64 for the polynomial regression model. The roughness values predicted using the models differ by at least 4 μm on average from the measured values. 

Figure 7b–e show the diagrams illustrating the fit between predicted and measured values (from the test set) for the four other models. The divergence between these values is shown by their wide alignment from the straight line, as reflected by a lower R^2^ coefficient value.

**Figure 8:**
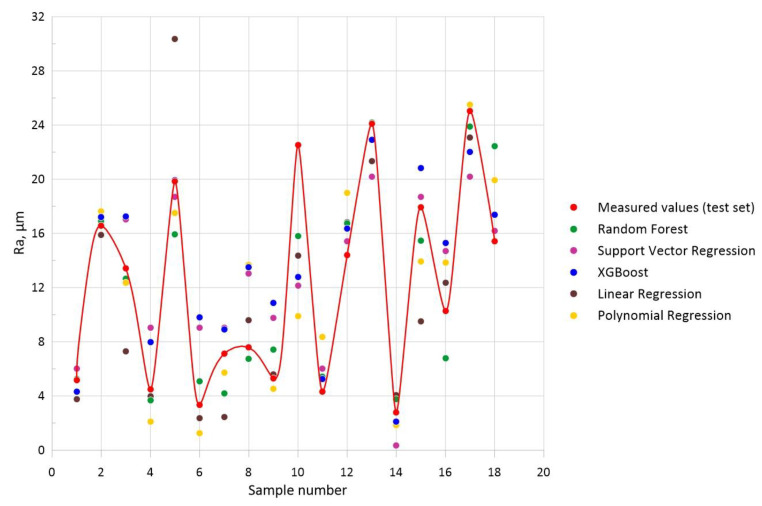
Comparison of the model-predicted and measured test set (connected by red line) values of roughness *Ra.*

For the visualization of the predicted values obtained with individual models, a diagram was created, shown in Figure 8. The obtained results were compared with the test set data. The quality of the models is visualized by the degree of their fit to the test values. The metrics from Table 4 are represented graphically.

**Figure 9:**
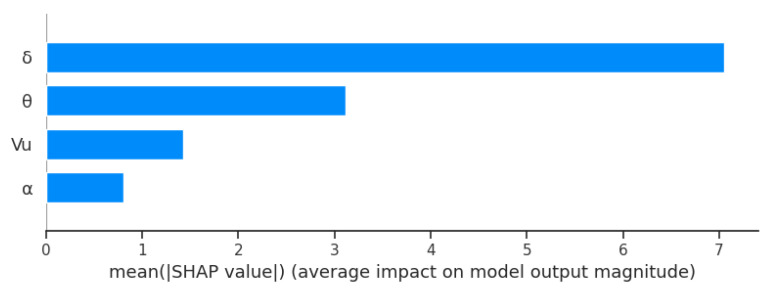
Variable significance plot for a random forest model.

Given the high effectiveness of the random forest model, the effect of individual input parameters on the roughness value predicted thereby was investigated. The SHAP library [32] was used to make it possible to present the effect of input variables and their values on the final result. Figure 9 shows, in descending order, the effect of the variables on the random forest model. The results demonstrate that the model is the most affected by the reduction ratio *δ*, whose Shapley value is more than twice as high as that of the successive parameter, i.e., the tool angle *θ*. Other key parameters for the model are the chuck velocity *V_u_* (its significance being twice lower than that of the *θ* angle) and the forming angle *α*, which describes the geometry of the tapered rollers.

**Figure 10:**
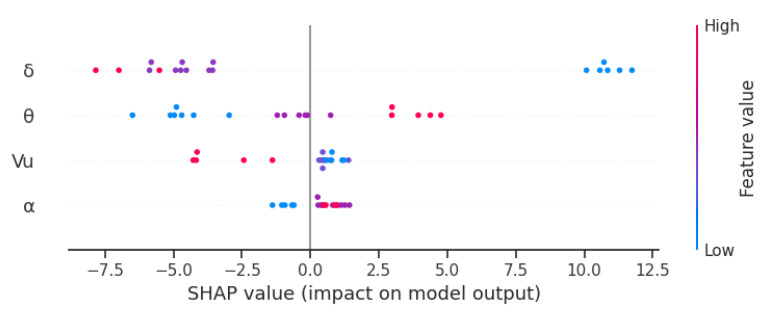
Effect of variables on roughness parameter for a random forest model.

Another SHAP library-based diagram (Figure 10) shows the effect of input variables on the obtained roughness of samples made of C60-grade steel. It can be observed that the low reduction ratio, i.e., *δ* = 1.13, causes an increase in *Ra.* On the other hand, an increase in the diameter reduction (higher *δ*) causes the *Ra* parameter to decrease, thus improving the outer surface quality of the product. Setting the tools at an angle *θ* of 7.5° increases the roughness parameter. A decrease in the *θ* angle leads to a lower *Ra* value. Increasing the chuck velocity *V_u_* from 10 mm/s to 40 mm/s also results in improved surface quality. As far as the forming angle is concerned, the use of the tapered rollers with *α* equal to 15° or 20° can ensure reduced product surface roughness.

## 4. Conclusions

This study demonstrated that an increase in the forming angle *α* caused an increase in the material roughness *Ra*. In effect, the external surface quality of products was reduced. In addition to that, the use of a higher tool angle *θ* relative to the rolling axis led to an increased surface roughness of the C60 steel samples. The use of a low axial velocity *V_u_* of the jaw chuck (10 mm/s) resulted in higher values of the roughness parameter *Ra*. With increasing the velocity, the roughness decreased. A similar relationship was observed when the reduction ratio *δ* was changed. An increase in the reduction ratio value (up to 1.53) led to a higher surface quality of produced parts.

The results made it possible to determine the optimal machine learning model for predicting the roughness of skew-rolled steel parts. A dataset consisting of the process setting parameters and target variable, i.e., the *Ra* parameter, was used. The data used for the models were obtained from experiments conducted with different settings, as well as from profilometric surface analysis. The models were based on regression in order to predict the continuous variable value. Based on the evaluation of the metrics, the random forest model was found to have the highest degree of fit (R^2^ = 0.84) for the training set. Also, it showed the lowest RMSE value of 2.98 μm. The other models, i.e., support vector regression, XGBoost, linear regression, and polynomial regression, yielded slightly lower fit values, ranging from 0.64 to 0.69. In addition, the effects of individual input variables and their interpretation using the random forest model on the final result were determined. For this purpose, the SHAP library was used.

The use of machine learning algorithms allows for more thorough interpretation of the phenomena occurring in skew rolling. The algorithms can be used to predict roughness values of steel parts, which will lead to increased efficiency of this rolling process because rolling parameters can be preselected, thus reducing the time required for preparatory work. This will have a positive effect on the final quality of the product surface, which can be important in the case of preforms because internal defects in forged parts can thus be prevented. The results of this study can be used to build a model with a high level of fit that can be implemented in a manufacturing environment. Nevertheless, it is recommended that the dataset be expanded by extending the experimental work. It is necessary to later verify the additionally introduced modifications on the prediction quality of machine learning models.

## Figures and Tables

**Table 1:** Parameter settings used in the experiments.

*α* (°)	*θ* (°)	*V_u_* (mm/s)	*δ* (-)
15 or 20 or 25	2.5	10	1.13
			1.3
			1.53
		20	1.13
			1.3
			1.53
		40	1.13
			1.3
			1.53
	5	10	1.13
			1.3
			1.53
		20	1.13
			1.3
			1.53
		40	1.13
			1.3
			1.53
	7.5	10	1.13
			1.3
			1.53
		20	1.13
			1.3
			1.53
		40	1.13
			1.3
			1.53

**Table 2:** Chemical composition of the C60-grade steel.

**C**	**Mn**	**Si**	**P**	**S**	**Cr**	**Ni**	**Cu**	**Mo**
0.59	0.66	0.23	0.013	0.027	0.1	0.09	0.2	0.02
**V**	**Al.**	**Ti**	**Sn**					
0.003	0.029	0.02	0.029					

**Table 3:** Mechanical properties of the C60-grade steel.

R_e_ (MPa)	R_m_ (MPa)	A_5_ (%)	Hardness (HB)
427	777	17.2	215

**Table 4:** Comparison of the metrics for regression model evaluation.

Model	MAE (μm)	RMSE (μm)	R^2^
Random Forest	2.29	2.98	0.84
Support Vector Regression	3.25	4.08	0.69
XGBoost	3.35	4.16	0.68
Linear Regression	3.17	4.37	0.65
Polynomial Regression	3.36	4.36	0.64

## Data Availability

Data are contained within the article.
